# The Dual Role of High Endothelial Venules in Cancer Progression versus Immunity

**DOI:** 10.1016/j.trecan.2020.10.001

**Published:** 2021-03

**Authors:** Stefan Milutinovic, Jun Abe, Andrew Godkin, Jens V. Stein, Awen Gallimore

**Affiliations:** 1 Infection and Immunity, School of Medicine, Cardiff University, Cardiff, UK; 2 Department of Oncology, Microbiology and Immunology, University of Fribourg, Fribourg, Switzerland

**Keywords:** high endothelial venules, cancer, immunity

## Abstract

Secondary lymphoid organs (SLOs) are important initiators and regulators of immunity. To carry out this function, the blood vasculature must deliver oxygen and nutrients and recruit circulating lymphocytes into the SLO parenchyma, where they encounter cognate antigen. High endothelial venules (HEVs) are specialised postcapillary venules that specifically serve this function and are found in all SLOs except spleen. It is becoming clear that alterations to HEV network density and/or morphology can result in immune activation or, as recently implicated, in providing an exit route for tumour cell dissemination and metastases. In this review, the structural plasticity of HEVs, the regulatory pathways underpinning this plasticity, and the relevance of these pathways to cancer progression will be discussed.

## Highlights

High endothelial venules (HEVs) are specialised blood vessels, found in secondary lymph nodes, where they play an important role in initiating and regulating immunity.
Recent studies indicate that HEVs can also provide an exit route for tumour cell dissemination and the establishment of metastases.
The dual roles of HEVs imply that they can both limit and accelerate progression of cancer.
It is important to address gaps in our understanding of HEV remodelling in order to uncover routes to promoting immune activation via HEV, whilst simultaneously limiting their ability to disseminate cancer cells.

## High Endothelial Venules (HEVs): Drivers of Immune Activation

HEVs comprise high endothelial cells (HECs) that are readily distinguished from other blood endothelial cells by their characteristic plump, cuboidal morphology, first described in the late 19th century [1]. Whilst they share common pan-endothelial cell (EC) markers such as CD31 and VE-cadherin [2], HECs preferentially express genes that are important for the role of HEVs in lymphocyte recruitment and immunological defence [3]. A key example is peripheral node addressin (PNAd), which acts as an adhesion molecule for CD62L (L-selectin)-expressing lymphocytes [3], an interaction that mediates the tethering and rolling of lymphocytes along HEVs, marking the initial stages of the multistep adhesion cascade, and ultimately leading to lymphocyte extravasation into the lymph node (LN) parenchyma [4,5]. The L-selectin/PNAd interaction is crucial for enabling naïve and central memory T cells as well as naïve B cells to home to LNs [4]. Ectopic HEVs have also been found at sites of chronic inflammatory diseases [6], infection [7], and, as described in detail in this review, in cancer [8]. In each of these settings, HEVs have been implicated in driving immune reactions, possibly by enabling homing of naïve immune cells to the diseased site [6., 7., 8.].

## HEV Remodelling in Reactive LNs

**Figure 1:**
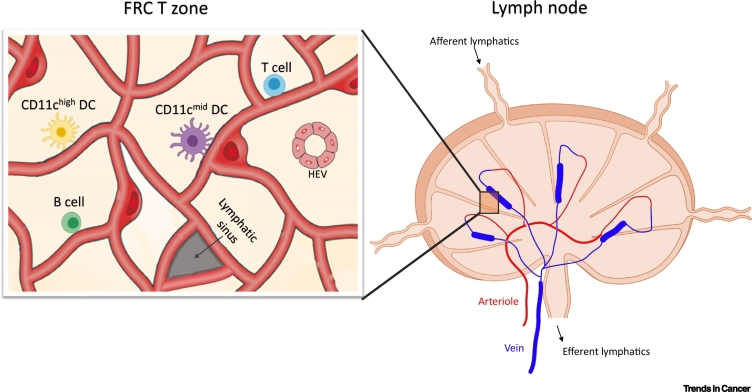
Vascular-Stromal Elements Involved in Lymph Node (LN) High Endothelial Venule (HEV) Remodelling. The LN feeding arteriole branches into capillaries, which in turn branch into postcapillary HEVs (thickened blue segment). Cross-section of vascular-stromal elements found in the T zone are depicted. HEVs are suspended in a reticular network characterised by collagen-rich fibrils ensheathed by reticular cells, termed fibroblastic reticular cells (FRCs), which form a network supporting immune cell migration and survival. In addition to FRCs, T cells, B cells, and dendritic cells (DCs) are involved in mediating HEV remodelling in response to ovalbumin in complete Freund’s adjuvant (OVA/CFA) immunisation. The initiation phase is driven by CD11c^Med^ DCs (purple), whilst the expansion phase is driven by T and B cells. CD11c^High^ DCs (yellow) are important in the resolution of this response. FRCs are also in close proximity to lymphatic vessels and may have a role in the regulation of this compartment as well. Figure adapted from [20,71].

Since the development, structure, and function of HEVs have been described in detail in previous reviews, a summary of the key features of HEV is described in Box 1. The functional and structural plasticity of HEVs in response to LN stimulation is also well documented. Alcian blue dye infusion, together with angiography, has been used to show extensive LN expansion and HEV remodelling in response to antigen stimulation [9,10]. This was characterised by an increase in HEV length and branching patterns [9,10] and accompanied by increased blood flow and lymphocyte trafficking [11]. Whilst these early studies revealed the contribution of EC proliferation in HEV plasticity, it has become clear that the co-operative activities of vascular, stromal [notably fibroblastic reticular cells (FRCs)], and immune cells are required for HEV remodelling and development of an immune response (Figure 1).

Box 1 **HEV Structure and Function**

In LNs, HEVs are localised to the cortical–paracortical junction and paracortex (where the T cell zone is located) and form part of the venular tree, a distinct hierarchy of venules that branch out from the largest collecting vein (order I) to the smallest postcapillary venules (order V) (Figure I) [74]. Intravital microscopy studies revealed that the major sites of lymphocyte recruitment occur in venule branches that are of the order III–V, which constitutes all HEVs [74] (Figure I). HECs have also been shown to exhibit tissue-specific specialisation [3]. Whole-genome expression profiling of HECs isolated from gut-associated lymphoid tissue (GALT) showed preferential expression of the enzyme β-galactoside α-2,6-sialyltransferase 1 (ST6Gal I), whilst HECs isolated from peripheral LNs had lower expression levels of this enzyme [3]. ST6Gal I was subsequently identified as a vascular addressin for the targeting of B cells to GALT [3]. Focal sites of recruitment are found within HEVs. Single-cell RNA sequencing has revealed that HECs under homeostatic conditions display heterogenous expression of several genes, including CCL21, within the same SLO [75]. This may reflect specialised functions of individual HECs and the establishment of preferential sites for immune cell transmigration (termed ‘exit ramps’) in different regions of the SLO (e.g., paracortex versus medulla) [76]. Furthermore, important differences in the regulation of genes controlling lymphocyte trafficking were also revealed by single-cell RNA sequencing [75]. Several genes involved in the synthesis of the 6-sulfo sLe^X^ epitope, which decorate PNAd, including the fucosyltransferase FucT-VII (Fut7) and core 2 branching enzyme Core2 GlcNAcT (Gcnt1), were found to require higher levels of LTβR-dependent signalling for expression than the sulfotransferase GlcNAc6ST-2 (Chst4) [75]. Such differential sensitivity of HEV genes to LTβR-dependent signalling may in part explain the cellular and spatial heterogeneity of peripheral LN HEVs [75].

**Figure I:**
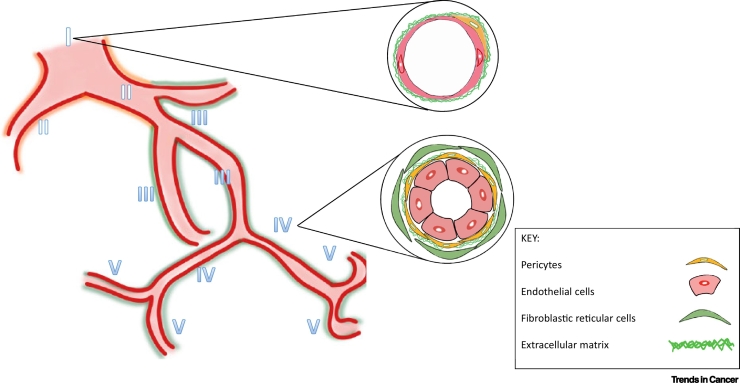
High Endothelial Venule (HEV) Structure and Localisation. The venular tree found in lymph nodes (LNs) is organised into a distinct hierarchy of branches that branch out from the collecting venule (order I) until the smallest postcapillary venules (order V). HEVs constitute all venules found on the order of III–V and have a distinct cuboidal morphology composed of plump high endothelial cells. HEVs display a thickened basal lamina surrounded by overlapping pericytes and are ensheathed by concentric layers of fibroblastic reticular cells. This is in contrast to normal venules, which consist of flat endothelial cells surrounded by a thin basal lamina and ensheathed by pericytes. Figure adapted from [4,77]

Alt-text: Box 1

HEV remodelling in response to immunisation with antigen [ovalbumin in complete Freund’s adjuvant (OVA/CFA)] has been extensively studied and is a well-regulated process that occurs in three distinct phases [12., 13., 14., 15., 16., 17.]. The initiation phase, which is T and B cell independent [16], lasts for 2 days and is driven by CD11c^+^ dendritic cells (DC) [14]. Upregulation of vascular endothelial growth factor (VEGF) by FRCs is in part stimulated by the release of IL-1β from CD11c^+^ DCs and monocytes [17]. This initiation phase is marked by increased VEGF-driven EC proliferation, FRC proliferation, and a modest increase in HEV EC numbers [14,16]. FRCs, which are normally tightly wrapped around vessels, stretch and begin to detach to allow for the second phase, characterised by vascular expansion, to occur [15]. During this phase, detachment of FRCs is mediated by CLEC-2 expressing CD11c^+^ cells [18], whilst podoplanin (PDPN) expression by FRCs is critical in the maintenance of overall HEV integrity during LN expansion and lymphocyte homing through its interaction with CLEC-2-expressing platelets [19]. In addition to EC and FRC proliferation, the expansion phase marks extensive increases in total HEV length and branching patterns [13] and lasts between days 2 and 5 [16]. This phase is strongly dependent on T and B cells [16], implying that HEV expansion reflects an effort to support the development of an adaptive immune response [20]. Recently, multicoloured fluorescent fate-mapping models, which enable labelling of adult LN ECs with specific colours to allow for subsequent progeny tracking at the single-cell level, have revealed that HECs act as local progenitors to create both capillaries and HEV neo-vessels during vascular expansion [21]. In addition to structural changes, HEVs revert to an immature phenotype (MAdCAM-1^high^HEC-6ST^low^) during this phase, which is caused by impaired afferent lymphatic vessel function [22], resulting in a dilution of DC-secreted LTβR ligands [23].

The vascular-stromal quiescence phase can last for several weeks and is mediated by CD11c^hi^ DCs [15]. This phase marks a reduction in HEV and FRC proliferation, reduction in HEV trafficking efficiency, and stabilisation of vessels by FRC reassembly around vessels [15]. HEVs also revert back to a mature phenotype during this period [22]. LN vascular expansion is then thought to eventually result in vascular regression following the resolution of the immune response [20]. Whilst both pre-existing and neo-vessels have been found to be pruned at the same pace during LN quiescence, the factors that induce such pruning remain to be identified [21].

The development of **mesoscopic imaging** techniques (see Glossary), such as **optical projection tomography (OPT)** and **light sheet fluorescence microscopy (LSFM)**, has allowed for the global analysis of HEV network remodelling in response to infection [24], immunisation, and autoimmune lymphadenopathy [13]. Such studies have revealed key similarities and differences between different LN stimulation settings (Box 2). The total number of vessels and branching points, including each individual vessel length and diameter, can be extracted from LSFM/OPT-acquired datasets (see Figure I in Box 2). Since vessel elongation and a reduction in diameter may result from vessel stretching or an increase in number of HECs (reflecting expansion), flow cytometric analysis and enumeration of HEC populations represent an important complement to 3D imaging [13,24]. Incorporation of these techniques into studies of LN remodelling should shed further light on the impact of alterations in HEV network/morphology on the course of antigen-specific immune responses in different settings.

Box 2 **3D Analysis of LN HEV Remodelling**

Lymphocytic choriomeningitis virus (LCMV) is a widely used mouse model for examining immune responses during viral infection due to it being noncytolytic and capable of inducing a wide range of immune responses, depending upon which LCMV strain is used [78]. LCMV has a strong tropism for SLOs and its impact on HEV remodelling was assessed using OPT [24]. LCMV and OVA/CFA immunisation shared several features, including extent of HEV branching (arborisation) and expansion, which likely reflect shared regulatory mechanisms underpinning HEV remodelling [13,24]. However, differences such as vessel arborisation preceding elongation during LCMV infection [24] likely reflect stimuli-specific differences. Indeed, LCMV-induced HEV remodelling is primarily driven by LTα_1_β_2_-expressing B cells and not by VEGF-A stimulation [24]. Therapeutic targeting of vessel remodelling can also be assessed by 3D imaging. For example, mesenchymal stem cells (MSCs) are well established inhibitors of inflammation and immunity and show therapeutic efficacy in several mouse models of human disease [79]. Whether MSC-mediated immunosuppression is driven by modulation of the LN vascular compartment was assessed using OPT [79]. MSC administration was shown to reduce HEV expansion following OVA/CFA immunisation, which lead to impaired immune cell trafficking [79]. Understanding how HEV remodelling progresses during different disease states will provide useful information for elucidating the factors controlling such changes and provide common targets for therapeutic targeting. By inducing HEV expansion it may be possible to promote immune activation by enhancing immune cell recruitment. Conversely, inhibiting HEV remodelling may prove useful for the treatment of diseases driven by excessive immune activation.

**Figure I:**
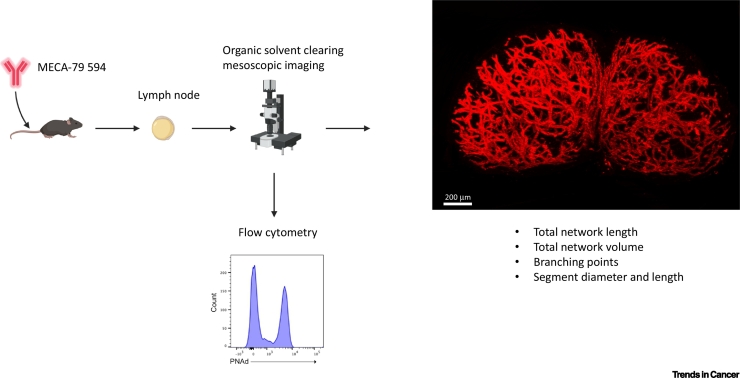
3D Imaging of High Endothelial Venule (HEV) Networks with Optical Projection Tomography (OPT) and Light Sheet Fluorescence Microscopy (LSFM). Whole lymph node (LN) HEV networks can be labelled intravenously using the fluorescently labelled MECA-79 antibody, which recognises the 6-sulpho sialyl Lewis X epitope presented on peripheral node addressin (PNAd). Labelled lymph nodes are optically cleared using organic solvents and imaged either by OPT or LSFM. **Extended volume confocal imaging** (EVIS imaging) can also be used to reconstruct the 3D structure of HEVs. Through the use of vessel tracing tools, several numerical parameters, including individual segment lengths and diameters, can be extracted from the 3D datasets. Following 3D imaging, samples can be rehydrated for flow cytometric analysis. Figure adapted from [24].

Alt-text: Box 2

## Sentinel LN HEV Remodelling

**Figure 2:**
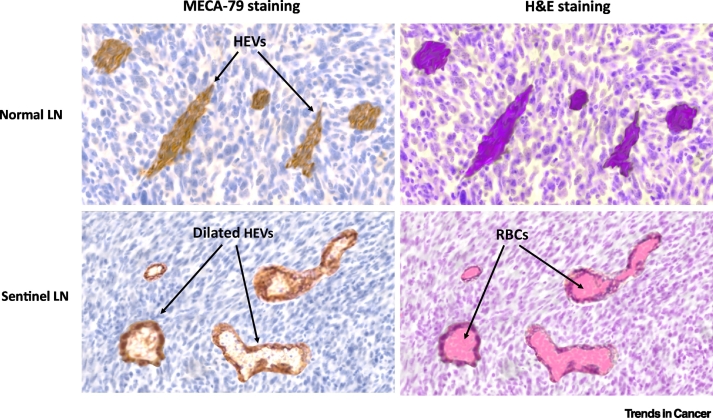
Sentinel Lymph Node (LN) High Endothelial Venule (HEV) Remodelling. Both murine and human sentinel LNs undergo extensive HEV remodelling. This is characterised by a thinning of the vessel wall and an increase in vessel diameter. A higher density of HEVs as well as the accumulation of red blood cells (RBCs) in the HEV lumen is associated with more aggressive disease and poorer survival. Functional changes to sentinel LN HEVs include reduced peripheral node addressin (PNAd) and CCL21 expression, leading to defective immune cell trafficking. Figure was illustrated in Adobe Photoshop 21.2 using an LN nuclear counterstain, which served as the background layer. All other objects, including the H&E stain, RBCs, and MECA-79 stain were illustrated using the background layer as a graphical template. Figure adapted from [27].

Regional LNs, which drain established tumours and are the first to receive metastatic cells (referred to as sentinel LN), are known to undergo tumour-reactive lymphadenopathy accompanied by both lymphatic and vascular expansion [25., 26., 27.]. Sentinel LN HEVs have been studied in the context of mouse models and human breast, squamous cell cancer, and melanoma and have been found to be remodelled even before metastatic tumour cells are detectable within the node [25., 26., 27., 28.]. Sentinel LN HEVs often exhibit loss of the functional HEV marker PNAd, comprise dilated lumens that are lined by a flat endothelium, and are engorged with red blood cells (RBCs) (Figure 2). Importantly, in human tongue squamous cell carcinoma, the patient’s overall survival (OS) risk was progressively worse as more sentinel LN HEVs were identified per high power field, with the highest risk seen in patients with dilated HEVs, with RBCs localised to the lumen [25]. This study showed that sentinel LN HEV remodelling is associated with a worse prognosis, regardless of whether LN metastasis is established [25].

The histological findings described earlier have been confirmed by electron microscopy imaging of metastatic sentinel LNs from oral and pharyngeal squamous cell carcinoma (OPSCC) patients, revealing the presence of remodelled HEVs engorged with RBCs displaying thin and dilated lumens with loose structure and noncontinuous basement membrane [28]. Real-time ultrasonography studies indicate that sentinel LNs have increased blood flow to large blood vessels [27]. Overall, these findings have led several researchers to speculate that sentinel LN HEV remodelling represents a skewing towards enhanced blood flow and diminished immune function [27].

## Signals Driving Sentinel LN Remodelling

Since alterations to sentinel LN HEVs are observed before tumour cells become detectable, it is conceivable that tumours may ‘prime’ sentinel LN vessels via lymph-borne tumour-specific or inflammatory mediators to prepare for the arrival of malignant cells. Indeed, there is evidence to suggest that lymph-borne factors as opposed to blood-borne factors stimulate HEV remodelling, as injecting dead tumour cells and plasma from tumour-bearing mice does not induce systemic LN HEV remodelling [27]. Furthermore, given the important role of the lymphatic vasculature in maintaining HEV function [22], alterations of lymphatic vessels following tumour establishment may lead to the delivery of such signalling cues that drive sentinel LN HEV remodelling. Indeed, tumour-secreted factors and extracellular vesicles have been shown to lead to the perturbation of ECs at distant sites, which is one the first steps in the establishment of a premetastatic niche (PMN) [29]. Tumour-derived exosomes released by melanoma cells have been shown to home specifically to the sentinel LN, resulting in the induction of proangiogenic factors, implicated in establishment of a PMN, which supports the recruitment and growth of metastatic melanoma cells [30].

In a murine model of VEGF-D-driven tumour metastasis, reduced expression of bone morphogenetic protein-4 (BMP-4) in HEVs was associated with a remodelling of HEVs towards a flat, thin-walled phenotype [31]. Whilst these findings indicate that BMP-4 may act as a molecular sign-post of HEV remodelling, its utility as a therapeutic target remains to be comprehensively explored [31]. In oesophageal cancer patients, transcriptomic profiling of metastasis-free regional LNs from patients with existing metastatic nodes revealed that Dickkopf-1 (DKK1), a Wnt antagonist, was the most significantly downregulated gene as compared with regional LNs from oesophageal cancer patients without metastatic nodes [32]. Interestingly DKK1, which is expressed by LN vascular ECs [32], has a negative effect on tumour angiogenesis [33] and perfusion [34]. The impact of DKK1 downregulation on HEV structure/function has not yet been examined.

## Sentinel LN Remodelling versus Immune Reactive LN Remodelling

Lymph-borne factors that drive sentinel LN remodelling may be distinct from those involved in immune reactive remodelling. In support of this, it has been shown that nude mice are susceptible to sentinel LN remodelling but resistant to endotoxin-induced LN remodelling, suggesting a lack of T cell involvement in the former [27]. In this study, sentinel LNs were characterised as comprising thin-walled, highly dilated HEVs with sites of RBC engorgement, contrasting with the immune reactive LNs that comprised dense lymphocyte-rich HEV networks. The findings of this study, which imply that immune reactive and sentinel LN remodelling are distinct processes, are supported by other observations relating to sentinel LN HEVs. Intravital microscopy of LNs draining B16 melanoma tumours revealed impaired lymphocyte recruitment along sentinel LN HEVs [35]. Lymphocyte adhesion in HEVs was found to be reduced, along with a decrease in expression of the chemokine CCL21 [35], which mediates lymphocyte arrest on the HEV endothelium [4]. Again, this occurred irrespective of LN metastasis, suggesting a disruption of HEV function in response to the primary tumour [35]. In a separate study examining established tumour nests within LN, PNAd was shown to be lost from parts of the HEV tracking from the tumour margin to the central portion of the tumour nests [27], suggesting that the HEVs further de-differentiate after integrating into the metastatic tumour vasculature [27].

Further studies are, however, required before it can be concluded that sentinel LN and immune reactive LN remodelling are distinct processes. Indeed, it is possible that immune cell activation in sentinel LNs [36] driven by tumour-derived cues could lead to HEV remodelling. Delivery of tumour antigens by DCs and/or tumour-driven production of inflammatory cytokines could serve as important HEV remodelling cues. The degree to which this occurs may be dependent on the type of cancer cell, the factors it releases, as well as its inherent immunogenicity. Whether sentinel LN HEV remodelling is accompanied by expansion and/or detachment of FRCs, which occur during immune reactive remodelling, is also not currently known [37].

## Sentinel LNs and Tumour Cell Dissemination

The involvement of LNs as a gateway for further dissemination of tumour cells is supported by correlative evidence from mouse models of breast and prostate cancer [38., 39., 40.] as well as human breast cancer patients who have worse outcomes if they present with lymphovascular invasion and nodal metastasis [41., 42., 43., 44.]. In support of this, regional LN irradiation has been associated with improved outcomes in early-stage breast cancer patients [45,46]. The ability of HEVs to provide a lymphatic-venous shortcut for metastasising cells to directly access the blood circulation has been suggested previously [37]. The finding that 20% of women with node-negative breast cancer still develop metastasis supports the existence of an alternative route for tumour cell dissemination, not reliant upon the stepwise progression of tumour cells from primary tumour to distal lymphaticovenous connections such as the thoracic duct [25].

Tumour cell dissemination via HEVs was confirmed recently by two studies. The first study demonstrated that the intralymphatic microinfusion of 4T1 mammary carcinoma tumour cells leads to the accumulation of 4T1 cells within the subcapsular sinus of the draining LN [47]. Three days postinfusion, 4T1 cells were found closely associated with HEVs and also intravasated the HEV lumen [47]. By infusing mCherry^+^ luciferase^+^ 4T1 cells, it was possible to detect metastasis in the lungs through the use of whole-animal *in vivo* bioluminescence imaging [47]. Lung metastases were only detectable 3 days after intralymphatic infusion, coinciding with the time taken for the tumour cells to intravasate HEVs [47]. Furthermore, the ligation of downstream efferent lymphatics did not compromise the ability of 4T1 cells to seed the lungs [47], confirming the role of HEVs as an active gateway for tumour cell dissemination. A separate study used several cancer cell lines (including 4T1, B16F10 melanoma, and SCCV2 squamous cell carcinoma) engineered to express the photoconvertible protein Dendra2 [48]. Dendra2 is a green-light emitting protein that, upon excitation with a 405-nm laser diode, is converted to a red-light emitting protein, making it possible to track the migratory fate of cells photoconverted at tissue-specific sites. By orthotopically implanting Dendra2-expressing tumour cells and photoconverting cells within the metastatic draining LN following tumour establishment, the presence of red-light emitting cells was detected in the systemic blood circulation and in the lungs [48]. Tumour cell migration towards LN blood vessels and subsequent migration within vessels was also confirmed by two-photon microscopy [48]. These two key studies therefore showed that HEVs are the main exit route by which tumour cells gain entry to the blood circulation.

Interestingly, tumour establishment was not required for HEVs to disseminate intralymphatic-infused 4T1 cells [47]. HEVs may, therefore, in their basal state (without prior remodelling), support tumour cell dissemination. Given that sentinel LN HEV remodelling is known to occur and precede tumour cell colonisation of the LN [27] and that increased HEV density along with the presence of RBCs is associated with reduced OS [25], it would be of interest to examine the impact this remodelling has on tumour cell dissemination efficiency.

What is currently lacking is a global analysis of sentinel LN HEV remodelling using the aforementioned 3D imaging techniques. Characterising the tumour-driven progression of HEV remodelling, and comparing this to previously characterised changes to HEV networks in immune reactive LNs, may reveal shared features that could be therapeutically targeted if such changes are indeed found to be important mediators of tumour metastasis.

## Ectopic HEVs in the Tumour Microenvironment

The presence of HEVs in primary human solid tumours and its association with lymphocyte infiltration has been shown in several tumour types, including breast, lung, ovarian, colon, and melanoma [8]. Importantly, the extent of lymphocyte infiltration was also found to be associated with favourable clinical outcome in several of these cancers, including ovarian carcinoma, lung, and colon cancer [49., 50., 51.]. HEVs can be found in isolation or as parts of lymphoid-like tissue, termed tertiary lymphoid structures (TLSs), which vary in their respective organisational capacity but are not encapsulated like LNs [52]. The role of TLS in cancer is further described in Box 3 and has been reviewed extensively elsewhere [52,53] but in general and with the information available to date, their presence in solid malignancies is associated with favourable prognosis [54,55].

Box 3 **Role of HEVs in Cancer**

In a retrospective cohort of 146 invasive breast cancer patients, HEVs were found to correlate with disease-free, metastasis-free, and overall survival [8]. In addition to tumour regression, the expression of both naïve T cell and Th1 genes correlated with HEV density in human melanoma, further supporting the role of HEVs as active sites of lymphocyte recruitment and activation [61]. In patients with oral squamous cell carcinoma, the presence of HEVs was associated with 5-year-longer disease specific survival (DSS) [62]. Interestingly higher-grade tumours (T3 and T4 stage) had less HEVs than lower grade tumours and the complete absence of HEVs was associated with worse DSS [62].

In keeping with their function in SLOs [56], several murine studies further support the role of intratumoural HEVs as active sites of lymphocyte recruitment [57,58,68,72,73]. For example, intravenously injected GFP^+^ splenocytes can be recruited to spontaneously induced TLS in a model of inflammation-driven carcinogenesis [72]. Furthermore, in a B16 melanoma model, the induction of TLS in splenectomised LTα^–/–^ mice, which lack all peripheral LNs, leads to the recruitment and induction of specific T cell responses, suggesting *in situ* priming at TLS sites [73]. Even in the absence of supporting TLSs, HEVs have been implicated in recruiting and initiating specific T cell responses [57,58,68].

Alt-text: Box 3

Unlike in secondary lymphoid organs (SLOs), however [56], the functional consequence of HEV formation in tumours remains to be determined by intravital microscopy [52]. HEVs may simply be a by-product of an ongoing immune response or actively involved in shaping the immune response through immune cell recruitment [52]. By depleting regulatory T cells (Tregs) in a fibrosarcoma tumour model, roughly 50% of tumours developed HEVs and led to high tumour-infiltrating lymphocyte (TIL) frequencies and improved control of tumour growth [57,58]. Importantly, by abrogating HEV development through TNFR signalling blockade, the TIL frequency was comparable with those tumours that do not develop HEVs following Treg depletion [57], suggesting an active role of HEVs in immune cell recruitment. Lastly, not all TLS/HEVs may result in improved antitumour immunity. B16-F10 melanoma tumours engineered to express CCL21 were reported to form TLSs that have an immunosuppressive role through the recruitment of Tregs [59]. Similarly, immunosuppressive activated Tregs were found localised to TLS in a mouse model of lung adenocarcinoma [60]. However, robust antitumour T cell responses and tumour destruction was achievable following local Treg depletion in the lung [60], suggesting that the role of TLS in tumour progression can be modulated by therapeutic intervention. Intratumoural HEVs have also been associated with worse prognosis. In OPSCC, two types of HEV were identified: a classical HEV phenotype associated with lymphocyte infiltrate, and an HEV-like phenotype characterised by thin-walled, dilated lumens containing RBCs, which were not associated with lymphocyte infiltrate but found adjacent to tumour cell clusters [28]. Whilst the density of intratumoural HEVs was found not to be significantly different between metastasis and nonmetastasis groups, a higher density of HEV-like vessels in the primary tumour was found to be associated with LN metastasis [28]. Whilst this suggests that the presence of HEV-like vessels in the tumour can lead to the dissemination of tumour cells, it is also important to note that widespread changes to sentinel LN HEVs were also described [28]. Clearly, further studies are needed to examine whether intratumoural HEVs, predominantly associated with an enhanced T cell infiltrate and tumour control, may, in certain situations, promote immunosuppression and even metastasis into and beyond the draining LNs.

## HEV Therapeutic Induction and Targeting

In spite of the aforementioned caveats, the correlation between HEV formation, increased lymphocyte infiltration, and more favourable prognosis in various cancer types [8,61,62] has provided a rationale for therapeutically inducing HEV formation in tumours. Several studies have implicated vessel normalisation and stabilisation as an important prerequisite for TLS formation [63., 64., 65.]. The combined use of checkpoint inhibitors (anti PD-L1) with antiangiogenic therapy has been shown to lead to the induction of HEVs in murine models of breast and pancreatic neuroendocrine mouse tumours but not in glioblastoma (GBM), which lacks a pre-existing activated cytotoxic T cell infiltrate [65]. However, administration of a LTβR agonist, together with anti-PD-L1 and anti-VEGF/VEGFR, led to the induction of HEVs and a reduced tumour burden [65]. Similarly, the selective targeting of LIGHT [a ligand that signals through LTβR and herpes virus entry mediator (HVEM)] to tumour vasculature via vascular targeting peptides (VTPs) also results in vessel normalisation and HEV induction [63]. When combined with antiangiogenic therapy and immune checkpoint blockade, LIGHT-VTP triple therapy was effective against GBM, permitting the induction of HEVs and recruitment of CD3^+^ tumour-infiltrating T cells, leading to a reduction in tumour growth burden [63]. Normalised vasculature may therefore permit immune cell trafficking whilst immune cell activation through immune checkpoint blockade leads to the release of cytokines, which stimulate HEV formation. This points to a reciprocal interaction between adaptive immunity and tumour vasculature, resulting in a positive feedback loop similar to the one described in a fibrosarcoma tumour model following Treg depletion and induction of HEV formation via TNFR signalling [57].

HEVs can also be exploited to effectively deliver chemotherapeutic drugs. Pancreatic ductal adenocarcinoma (PDAC) is a lethal disease characterised by a dense stroma and extensive desmoplastic reaction, which limits efficient drug delivery [66]. Targeting ectopic HEVs with MECA79-Taxol-nanoparticles was recently showed to improve Taxol responses through PDAC growth suppression [66]. Furthermore, it was recently reported that preoperative neoadjuvant chemoradiotherapy leads to a higher density of tertiary lymphoid organ-containing HEVs, which are associated with a slightly better prognosis [67]. This provides a rationale to both induce HEVs and exploit them for effective chemotherapeutic drug delivery in PDAC treatment.

## Concluding Remarks

**Figure 3:**
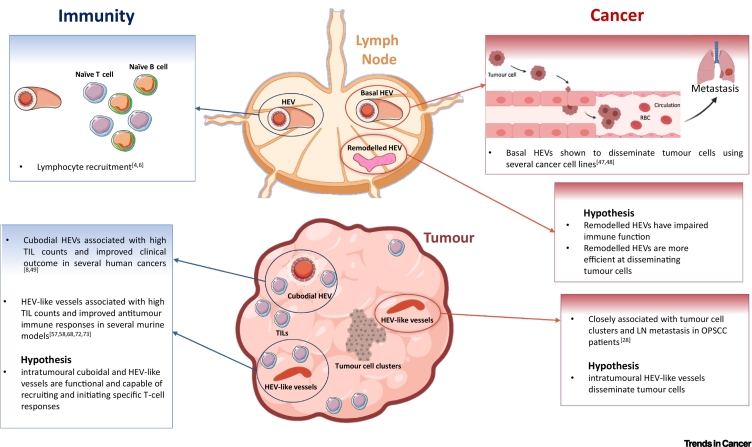
Dual Roles of High Endothelial Venules (HEVs) in Cancer Progression and Immunity. The dual roles of HEVs as initiators of immunity and as a major site for tumour cell dissemination in both lymph nodes and tumours is summarised. Whilst lymphocyte recruitment and tumour cell dissemination via basal HEVs has been demonstrated via 2-photon imaging, the functional role of remodelled HEVs in the lymph node has yet to be determined. Similarly, whilst intratumoural HEVs have been associated with high tumour-infiltrating lymphocyte (TIL) counts as well as lymph node (LN) metastasis in oral and pharyngeal squamous cell carcinoma (OPSCC) patients, their functional role has not been directly examined by intravital imaging. See [4,5,8,28,47., 48., 49.,^57^,^58^,^68^,^72^,^73^].

The dual role of HEVs as promoters of tumour cell dissemination in naïve/remodelled sentinel LNs and as drivers of effective antitumour immunity in cancer (Figure 3) raises several important questions (see Outstanding Questions). Firstly, HEV-inducing agents may simultaneously drive antitumour immunity whilst promoting tumour cell dissemination by remodelling sentinel LNs. The functional consequences of sentinel LN HEV remodelling should be examined as well as the regulatory mechanisms that drive such changes. Furthermore, progressive structural changes to HEVs as a consequence of various HEV-inducing therapies should be mapped, ideally by 3D imaging.

The signalling pathways involved in SLO formation and maintenance are largely overlapping and selective targeting of HEV neogenesis is therefore an important factor to consider [52]. However, the functional impact selective HEV-inducing pathways exert on sentinel LNs have not been explored. For example, the TNFR signalling pathway has been implicated in driving the formation of HEVs in a murine fibrosarcoma tumour model [57] as well as in models of melanoma and lung carcinoma [68]. In the fibrosarcoma model, Treg depletion was found to be a prerequisite for licencing HEV formation [57]. However, Treg depletion was found to lead to a disruption of LN HEV morphology [57]. This was characterised by a more open HEV lumen, the functional consequences of which have not been further explored. Such changes share features with the tumour carrier phenotype seen in sentinel LN HEVs and warrant further investigation. If this promotes tumour cell dissemination, then tumour selective drug delivery (such as the use of VTPs) may be required. VTPs are short peptides (5–9 amino acids long), which home specifically to neovessels found in either dysplastic lesions or in malignant tumours [69].

Whether intratumoural HEVs can support tumour cell dissemination is also currently not known. The functional consequence of intratumoural HEVs as both immune cell carriers and exit routes for tumour cells should be explored. In support of a tumour cell disseminating role, the presence of HEV-like vessels in OPSCC primary tumours was associated with sentinel LN metastasis [28]. Alternatively, intratumoural HEV remodelling may mark the resolution of an immune response. In human primary cutaneous melanoma, flat HEVs were found to be associated with tumour regression, whilst cuboidal HEVs were associated with lymphocyte infiltration [70]. Similarly, flat-walled HEVs in oral squamous cell carcinoma patients were associated with low-grade inflammation, whilst cuboidal HEVs were associated with lymphocyte infiltration [62]. The association of specific HEV phenotypes with tumour progression and/or LN metastasis was not examined in these studies. Further work is needed to address the regulatory mechanisms driving intratumoural HEV remodelling and the resulting functional implications.

Lastly, do the progressive changes of HEVs during sentinel remodelling mark the loss of immune cell recruiting functionality, leading to an impairment in immune activation? If so, then how might it be possible to drive remodelling towards an immune cell carrier instead of tumour cell carrier? There is evidence that resection of sentinel LNs leads to a significant reduction in metastatic spread but evidence also points to an important role for sentinel LNs in the induction of effective antitumour immune responses [36]. A better understanding of LN remodelling under different conditions is therefore warranted, as this may open up novel avenues for promoting immune activation whilst simultaneously limiting cancer cell dissemination.

Outstanding Questions What are the factors that drive sentinel lymph node (SLN) HEV remodelling?

Does SLN HEV remodelling lead to impaired immune activation?

Are SLN HEVs more efficient at disseminating tumour cells than basal LN HEVs?

What impact do HEV-inducing therapies have on LN HEV networks and how might this affect their role as both regulators of immunity and sites for tumour cell dissemination?

Are intratumoural HEVs capable of disseminating tumour cells and how might this be further affected by HEV-inducing therapies?

Alt-text: Outstanding Questions

## Glossary

**Extended volume confocal imaging**: a confocal microscopy technique that uses a high precision milling device to remove the outermost surface of the sample once it has been imaged. This technique uses a serial block-face imaging approach to overcome the penetration depth limit associated with laser scanning microscopy.
**Light sheet fluorescence microscopy (LSFM)**: a mesoscopic imaging technique that utilises a thin light sheet positioned perpendicular to the detection objective to achieve selective illumination of the focal plane. This allows for rapid imaging of thick specimens with low photobleaching and phototoxic effects.
**Mesoscopic imaging**: imaging techniques that probe the structures of whole samples (mm scale) typically for objects on the order of 1–10 mm, which are too large to handle with conventional widefield, confocal, or 2-photon microscopy.
**Optical projection tomography (OPT)**: a mesoscopic imaging technique that can acquire isotropic light or fluorescent images of a sample as it is being rotated by a stepper motor through a full 360° rotation. OPT uses low numerical aperture objectives that provide the large depth of field and working distance needed to achieve a 3D rendering of the specimen.

## References

[1] Thomé R. (1898). Endothelien als phagocyten (aus den lymphdrüsen von Macacus cynomolgus). Arch. Mikrosk. Anat..

[2] Pfeiffer F. (2008). Distinct molecular composition of blood and lymphatic vascular endothelial cell junctions establishes specific functional barriers within the peripheral lymph node. Eur. J. Immunol..

[3] Lee M. (2014). Transcriptional programs of lymphoid tissue capillary and high endothelium reveal control mechanisms for lymphocyte homing. Nat. Immunol..

[4] Girard J-P. (2012). HEVs, lymphatics and homeostatic immune cell trafficking in lymph nodes. Nat. Rev. Immunol..

[5] Miyasaka M., Tanaka T. (2004). Lymphocyte trafficking across high endothelial venules: dogmas and enigmas. Nat. Rev. Immunol..

[6] Aloisi F., Pujol-Borrell R. (2006). Lymphoid neogenesis in chronic inflammatory diseases. Nat. Rev. Immunol..

[7] Neyt K. (2012). Tertiary lymphoid organs in infection and autoimmunity. Trends Immunol..

[8] Martinet L. (2011). Human solid tumors contain high endothelial venules: association with T- and B-lymphocyte infiltration and favorable prognosis in breast cancer. Cancer Res..

[9] Herman P.G. (1972). Blood microcirculation in the lymph node during the primary immune response. J. Exp. Med..

[10] Anderson N.D. (1975). Microvascular changes in lymph nodes draining skin allografts. Am. J. Pathol..

[11] Hay J.B., Hobbs B.B. (1977). The flow of blood to lymph nodes and its relation to lymphocyte traffic and the immune response. J. Exp. Med..

[12] Chyou S. (2008). Fibroblast-type reticular stromal cells regulate the lymph node vasculature. J. Immunol..

[13] Kumar V. (2012). Optical projection tomography reveals dynamics of HEV growth after immunization with protein plus CFA and features shared with HEVs in acute autoinflammatory lymphadenopathy. Front. Immunol..

[14] Webster B. (2006). Regulation of lymph node vascular growth by dendritic cells. J. Exp. Med..

[15] Tzeng T.-C. (2010). CD11c(hi) dendritic cells regulate the re-establishment of vascular quiescence and stabilization after immune stimulation of lymph nodes. J. Immunol..

[16] Chyou S. (2011). Coordinated regulation of lymph node vascular-stromal growth first by CD11c+ cells and then by T and B cells. J. Immunol..

[17] Benahmed F. (2014). Multiple CD11c+ cells collaboratively express IL-1beta to modulate stromal vascular endothelial growth factor and lymph node vascular-stromal growth. J. Immunol..

[18] Acton S.E. (2014). Dendritic cells control fibroblastic reticular network tension and lymph node expansion. Nature.

[19] Herzog B.H. (2013). Podoplanin maintains high endothelial venule integrity by interacting with platelet CLEC-2. Nature.

[20] Dasoveanu D.C. (2016). Regulation of lymph node vascular-stromal compartment by dendritic cells. Trends Immunol..

[21] Mondor I. (2016). Clonal proliferation and stochastic pruning orchestrate lymph node vasculature remodeling. Immunity.

[22] Liao S., Ruddle N.H. (2006). Synchrony of high endothelial venules and lymphatic vessels revealed by immunization. J. Immunol..

[23] Moussion C., Girard J-P. (2011). Dendritic cells control lymphocyte entry to lymph nodes through high endothelial venules. Nature.

[24] Kumar V. (2010). Global lymphoid tissue remodeling during a viral infection is orchestrated by a B cell-lymphotoxin-dependent pathway. Blood.

[25] Lee S.Y. (2012). 2011 Young surgeon’s award winner: high endothelial venules: a novel prognostic marker in cancer metastasis and the missing link?. Ann. Acad. Med. Singap..

[26] Chung M.K. (2012). Lymphatic vessels and high endothelial venules are increased in the sentinel lymph nodes of patients with oral squamous cell carcinoma before the arrival of tumor cells. Ann. Surg. Oncol..

[27] Qian C-N. (2006). Preparing the “soil”: the primary tumor induces vasculature reorganization in the sentinel lymph node before the arrival of metastatic cancer cells. Cancer Res..

[28] Shen H. (2014). Alterations of high endothelial venules in primary and metastatic tumors are correlated with lymph node metastasis of oral and pharyngeal carcinoma. Cancer Biol. Ther..

[29] Peinado H. (2017). Pre-metastatic niches: organ-specific homes for metastases. Nat. Rev. Cancer.

[30] Hood J.L. (2011). Exosomes released by melanoma cells prepare sentinel lymph nodes for tumor metastasis. Cancer Res..

[31] Farnsworth R.H. (2011). A role for bone morphogenetic protein-4 in lymph node vascular remodeling and primary tumor growth. Cancer Res..

[32] Otto B. (2014). Molecular changes in pre-metastatic lymph nodes of esophageal cancer patients. PLoS One.

[33] Saupe F. (2013). Tenascin-C downregulates wnt inhibitor dickkopf-1, promoting tumorigenesis in a neuroendocrine tumor model. Cell Rep..

[34] Park H. (2014). Distinct roles of DKK1 and DKK2 in tumor angiogenesis. Angiogenesis.

[35] Carriere V. (2005). Cancer cells regulate lymphocyte recruitment and leukocyte-endothelium interactions in the tumor-draining lymph node. Cancer Res..

[36] Maeda T. (2018). Immune-mediated antitumor effect of a transplanted lymph node. Int. J. Cancer.

[37] Qian C-N. (2007). Prospects for vasculature reorganization in sentinel lymph nodes. Cell Cycle.

[38] Roberts N. (2006). Inhibition of VEGFR-3 activation with the antagonistic antibody more potently suppresses lymph node and distant metastases than inactivation of VEGFR-2. Cancer Res..

[39] Burton J.B. (2008). Suppression of prostate cancer nodal and systemic metastasis by blockade of the lymphangiogenic axis. Cancer Res..

[40] Chen Z. (2005). Down-regulation of vascular endothelial cell growth factor-C expression using small interfering RNA vectors in mammary tumors inhibits tumor lymphangiogenesis and spontaneous metastasis and enhances survival. Cancer Res..

[41] Nathanson S.D. (2010). The role of lymph node metastasis in the systemic dissemination of breast cancer. Indian J. Surg. Oncol..

[42] Jatoi I. (1999). Significance of axillary lymph node metastasis in primary breast cancer. J. Clin. Oncol..

[43] Mohammed R.A.A. (2007). Improved methods of detection of lymphovascular invasion demonstrate that it is the predominant method of vascular invasion in breast cancer and has important clinical consequences. Am. J. Surg. Pathol..

[44] Clarke M. (2005). Effects of radiotherapy and of differences in the extent of surgery for early breast cancer on local recurrence and 15-year survival: an overview of the randomised trials. Lancet (London, England).

[45] Poortmans P.M. (2015). Internal mammary and medial supraclavicular irradiation in breast cancer. N. Engl. J. Med..

[46] Whelan T.J. (2015). Regional nodal irradiation in early-stage breast cancer. N. Engl. J. Med..

[47] Brown M. (2018). Lymph node blood vessels provide exit routes for metastatic tumor cell dissemination in mice. Science.

[48] Pereira E.R. (2018). Lymph node metastases can invade local blood vessels, exit the node, and colonize distant organs in mice. Science.

[49] Galon J. (2006). Type, density, and location of immune cells within human colorectal tumors predict clinical outcome. Science.

[50] Pages F. (2005). Effector memory T cells, early metastasis, and survival in colorectal cancer. N. Engl. J. Med..

[51] Pages F. (2009). In situ cytotoxic and memory T cells predict outcome in patients with early-stage colorectal cancer. J. Clin. Oncol..

[52] Colbeck E.J. (2017). Tertiary lymphoid structures in cancer: drivers of antitumor immunity, immunosuppression, or bystander sentinels in disease?. Front. Immunol..

[53] Sautes-Fridman C. (2019). Tertiary lymphoid structures in the era of cancer immunotherapy. Nat. Rev. Cancer.

[54] Cabrita R. (2020). Tertiary lymphoid structures improve immunotherapy and survival in melanoma. Nature.

[55] Helmink B.A. (2020). B cells and tertiary lymphoid structures promote immunotherapy response. Nature.

[56] von Andrian U.H., Mempel T.R. (2003). Homing and cellular traffic in lymph nodes. Nat. Rev. Immunol..

[57] Colbeck E.J. (2017). Treg depletion licenses T cell-driven HEV neogenesis and promotes tumor destruction. Cancer Immunol. Res..

[58] Hindley J.P. (2012). T-cell trafficking facilitated by high endothelial venules is required for tumor control after regulatory T-cell depletion. Cancer Res..

[59] Shields J.D. (2010). Induction of lymphoidlike stroma and immune escape by tumors that express the chemokine CCL21. Science.

[60] Joshi N.S. (2015). Regulatory T cells in tumor-associated tertiary lymphoid structures suppress anti-tumor T cell responses. Immunity.

[61] Martinet L. (2012). High endothelial venules (HEVs) in human melanoma lesions: major gateways for tumor-infiltrating lymphocytes. Oncoimmunology.

[62] Wirsing A.M. (2016). Presence of tumour high-endothelial venules is an independent positive prognostic factor and stratifies patients with advanced-stage oral squamous cell carcinoma. Tumour Biol..

[63] He B. (2018). Vascular targeting of LIGHT normalizes blood vessels in primary brain cancer and induces intratumoural high endothelial venules. J. Pathol..

[64] Johansson-Percival A. (2017). De novo induction of intratumoral lymphoid structures and vessel normalization enhances immunotherapy in resistant tumors. Nat. Immunol..

[65] Allen E. (2017). Combined antiangiogenic and anti-PD-L1 therapy stimulates tumor immunity through HEV formation. Sci. Transl. Med..

[66] Bahmani B. (2018). Ectopic high endothelial venules in pancreatic ductal adenocarcinoma: a unique site for targeted delivery. EBioMedicine.

[67] Kuwabara S. (2019). Prognostic relevance of tertiary lymphoid organs following neoadjuvant chemoradiotherapy in pancreatic ductal adenocarcinoma. Cancer Sci..

[68] Peske J.D. (2015). Effector lymphocyte-induced lymph node-like vasculature enables naive T-cell entry into tumours and enhanced anti-tumour immunity. Nat. Commun..

[69] Hoffman J.A. (2003). Progressive vascular changes in a transgenic mouse model of squamous cell carcinoma. Cancer Cell.

[70] Avram G. (2013). The density and type of MECA-79-positive high endothelial venules correlate with lymphocytic infiltration and tumour regression in primary cutaneous melanoma. Histopathology.

[71] Fletcher A.L. (2015). Lymph node fibroblastic reticular cells in health and disease. Nat. Rev. Immunol..

[72] Di Caro G. (2014). Occurrence of tertiary lymphoid tissue is associated with T-cell infiltration and predicts better prognosis in early-stage colorectal cancers. Clin. Cancer Res..

[73] Schrama D. (2008). Immunological tumor destruction in a murine melanoma model by targeted LTalpha independent of secondary lymphoid tissue. Cancer Immunol. Immunother..

[74] von Andrian U.H. (1996). Intravital microscopy of the peripheral lymph node microcirculation in mice. Microcirculation.

[75] Veerman K. (2019). Single-cell analysis reveals heterogeneity of high endothelial venules and different regulation of genes controlling lymphocyte entry to lymph nodes. Cell Rep..

[76] Bajenoff M. (2006). Stromal cell networks regulate lymphocyte entry, migration, and territoriality in lymph nodes. Immunity.

[77] Ager A. (2017). High endothelial venules and other blood vessels: critical regulators of lymphoid organ development and function. Front. Immunol..

[78] Oldstone M.B.A. (2016). An odyssey to viral pathogenesis. Annu. Rev. Pathol..

[79] Zanotti L. (2016). Mouse mesenchymal stem cells inhibit high endothelial cell activation and lymphocyte homing to lymph nodes by releasing TIMP-1. Leukemia.

